# The impact of environmental regulation on green investment efficiency of thermal power enterprises in China-based on a three-stage exogenous variable model

**DOI:** 10.1038/s41598-024-58396-x

**Published:** 2024-04-10

**Authors:** Fang-rong Ren, Tao-feng Wu, Yang-jun Ren, Xiao-yan Liu, Xiaomei Yuan

**Affiliations:** 1 College of Economics and Management, Nanjing Forestry University, Nanjing, 210037 People’s Republic of China; 2 Changzhou Vocational Institute of Textile and Garment, Changzhou, 213164 People’s Republic of China; 3 Shaanxi Rural Revitalization Institute, Xi’an Innovation College of Yan’an University, Xi’an, 710100 People’s Republic of China

**Keywords:** Environmental regulation, Green investment efficiency, Thermal power enterprises, Three-stage model, Exogenous variable, Energy and society, Environmental economics, Sustainability

## Abstract

Due to the increased frequency of extreme weather events and the implementation of the China’s dual-carbon target, thermal power companies have been under pressure to construct green infrastructure and to actively pursue low-carbon transformation in response to stricter environmental regulations. This research thus selects 30 listed thermal power enterprises in China as study objects and assesses their green investment efficiency in the low-carbon transition process using three-stage DEA evaluation model with environmental regulation as an exogenous variable. Based on this, a benchmark regression model is used to corroborate the relationship between environmental regulation and green investment. Simultaneously, we carry out analysis to compare the correlation between thermal power firms’ green investment efficiency and their focus on green investments. The results show in terms of total efficiency that environmental regulation significantly improves the total efficiency of 80% of thermal power enterprises compared to the absence of this exogenous variable. With the addition of environmental regulation, firms’ total efficiency declines gradually in general from 2018 to 2022, with the mean value of efficiency falling by 0.068. In terms of stage-specific efficiency, the efficiency of the green investment stage of the majority of firms is between 0.3 and 0.6, which is much lower than that of the operational stage and the market performance stage. In terms of sub-indicator efficiency, both green investment efficiency and social donation efficiency among thermal power enterprises show obvious polarization, with 30% of them having an efficiency of 1 and 30% less than 0.1. In terms of green investment focus, thermal power unit renovation has a more obvious role in boosting the green investment efficiency of thermal power enterprises than do wind power and photovoltaic projects. Therefore, both governmental departments and thermal power enterprises need to take active measures in order to achieve green transformation from the perspective of green investment efficiency. Through the segmentation of important projects of green investment, this paper provides a reasonable investment direction reference for the sustainable transformation of China’s thermal power industry. It also provides a rich and novel theoretical basis for the Chinese government to further improve the relevant environmental protection laws and regulations of thermal power industry.

## Introduction

With environmental degradation and resource depletion becoming key impediments to global economic development, the green transformation of energy firms is on the horizon. According to the Energy Institute of the United Kingdom’s 2023 Statistical Yearbook of World Energy, worldwide energy demand increased by 1% in 2022. The historic increase of renewable energy has not altered the dominance of fossil fuels, which continue to supply 82% of global energy. Governments and other international organizations, such as the World Health Organization and the United Nations Environment Programme, have been actively encouraging research into environmental pollution and its effects on development in this respect. With coordinated efforts, the share of renewable energy in the world’s energy consumption from 2021 to 2022 would be 7.5%, or up around 1% from the year before. Indeed, 84% of the increase in net electricity consumption is met by a record 12% rise in wind and solar power generation.

Thermal power generation in China has accounted for 66.6% of total power generation in 2022, or growing 1.4% year on year. As a key energy supplier, thermal power firms must undergo green transformation in order to achieve sustainable development, and achieving this goal necessitates significant green investments. Global green investment increased from US$7 billion in 2000 to US$154 billion in 2010^1^, with the majority of that growth occurring in China. According to the United Nations Framework Convention on Climate Change (UNFCC), by 2050, $125 trillion in green investments will be needed to achieve carbon neutrality. According to the United Nations climate change annual report 2021, China is the largest contributor to green investment, particularly in the energy sector, valued at $266 billion. Among them, China's thermal power industry has also made large breakthroughs in the field of green investment. For example, according to the latest analysis of the China Electricity Council, the installed non-fossil energy generation capacity of the thermal power industry exceeded the size of the installed thermal power capacity for the first time in 2023, accounting for the first time for the proportion of the total installed capacity to exceed 50%.

To promote green investment, the China government has also continued to improve the environmental regulatory system by levying environmental protection taxes and opening up the carbon emissions trading market and green subsidy system. China is gradually forming a comprehensive environmental regulatory system combining command and control, market-led, and public participation^2,3^. According to the China Electricity Council, between 2016 and 2021 the power industry has already reduced carbon dioxide emissions by approximately 21.51 billion tons through non-fossil energy sources, reduced coal usage in power supply, and lower line-loss rate. China has now constructed the world’s largest ultra-low emission thermal power plant cluster.

**Figure 1:**
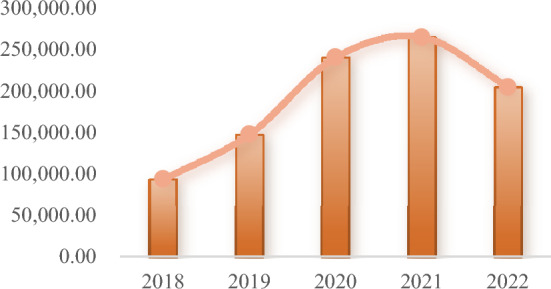
China’s thermal power industry annual green investment.

As thermal power firms are critical to energy savings and emission reduction, green investment can help drive their sustainable development and also meet market and government requirements by enhancing energy cleanliness and low carbon. Thermal power companies typically boost their green investments in the early stages of tighter environmental rules in order to enhance their brand value and company image. However, because pollution control costs are rising and profit margins are narrowing due to stricter environmental restrictions, green investments have decreased rather than increased (Fig. 1). Therefore, it is necessary for this study to dig deeper into the reasons behind the phenomenon—that is, the trade-off between costs and benefits of green investment in thermal power enterprises as well as the relationship between enterprise green investment efficiency and the choice of green investment focus.

Summarizing the research conducted by scholars at home and abroad in this field, there are three main limitations. First, the analysis of existing research in this field mainly focuses on developed regions or areas, and lacks research on developing countries^1,4^. However, the impact of environmental regulation on green investment has a strong geographical nature, and the differences in policies across countries can lead to different implementation strengths of environmental regulation by local governments. Second, due to the difficulty of obtaining data on corporate green investment, scholars' studies on green investment have mainly focused on the national or city cluster level, and lacked analysis on the corporate level^5,6^. Even the studies on corporate green investment are too broad in terms of industries, ignoring the fact that the impact of environmental regulations on green investment is limited by industry attributes^3,7^. Third, most of the existing literature uses the amount of green investments made by firms or cities, which is rather one-sided^8–11^. The input–output indicator system constructed with multiple variables is more comprehensive to measure the efficiency of green investment.

Therefore, this study overcomes the above shortcomings with the following three main contributions. First, it considers that changes in both the ESG index and media attention affect the financial support received by enterprises, which in turn influences the size of the funds used by enterprises for green investment projects. Therefore, this study incorporates the enterprise ESG index and media attention into the index system for assessing the green investment efficiency of thermal power enterprises.

Second, there are fewer studies on the green investment efficiency of energy enterprises in developing countries, especially Chinese thermal power enterprises. This study analyzes the impact of environmental regulations on the green investment efficiency of Chinese thermal power enterprises in the context of the country’s social system, taking into account the reality of its economic development.

Third, other studies have analyzed the efficiency of green investment using mainly econometric models, which can only deal with a single efficiency assessment. This study adopts the DEA model, which is not only able to deal with multiple inputs and multiple outputs, but also avoids subjectivity due to the assignment of weights.

## Literature review

### Impact of green investment

The literature on the impact of green investment of thermal power companies on their market value is divided into two main views: the promotion theory and the inhibition theory. Green investment is a form of resource allocation in thermal power companies, which can improve their environmental quality and corporate reputation by reducing the necessary investment in generating pollutants in their operations^1^. Taking listed energy companies in China as an example, some scholars have found that green investment improves their environmental performance through the reduction of environmental violations. This enhances the impact of green investment on the long-term market value of energy companies^12^. In addition, incorporating green investments into energy business strategy formulation helps reduce corporate environmental risks and prompts companies to create value in terms of social sustainability, ultimately leading to an increase in their market value^7,13,14^.

Some scholars have put forward opposite views on the impact of green investment on firm value, arguing that green investment has a negative or zero impact on firm performance. This is because green investment is relatively costly, takes away resources that companies use for normal production and operations, and usually requires a long payback period. Therefore, when enterprises conduct green investment operations, they incur a certain negative impact on cash flow and financial performance^15^. In short, green investment may have a certain inhibitory effect on the financial performance of enterprises. However, in the long term, green investments can help improve the environmental performance of energy companies, which in turn contributes to financial performance. Therefore, energy companies need to weigh inputs and outputs when making green investments as part of a long-term development strategy to achieve sustainable development.

### Factors affecting green investment

The literature on green investment has a variety of research angles. Some scholars have studied the influencing factors and development trends of green investment in the energy industry in developed countries by using econometric models from the perspective of macroeconomics^5,9^. The results show that green investment is influenced by economic growth, interest rates, and fuel prices^1^. At the same time, policy intervention of the national government, such as an environmental tax burden, also has a positive impact on green investment^10,11^. In view of the impact of an environmental tax burden, some scholars have used a variety of advanced panel data methods to conduct more detailed research. The results show although taxes have a weak catalytic effect compared to other factors that well-structured environmental regulation can still significantly promote green investment by firms^6^. Therefore, governments can provide tax incentives for companies to undergo green innovation, so that companies can get help with innovative projects.

Some scholars have studied the relationship between corporate ESG ratings and green investment using linear mixed models. The results show that there is a positive correlation between green investment and corporate ESG ratings, which has a significant impact on corporate sustainable operations^8^. In addition, some studies start from the perspective of public attention. Through the use of panel data and econometric models, research has found that strict environmental regulations prompt enterprises to increase green investment^5^. Given the high energy intensity of countries in the Asia–Pacific region, some economists have examined the relationship between green investment and renewable energy deployment from a renewable energy perspective. Using Panel Pooled Mean Group (PMG) technique, the study found that green investment potential positively affects renewable energy deployment^16^. Finally, from the perspective of R&D and innovation, some scholars use multiple regression models to conduct empirical analyses, and the results show that environmental regulation, by increasing green investment, not only benefits green innovation in the region, but also contributes to the neighboring region's^17^.

No matter from which viewpoint, scholars’ analysis of green investment mainly focuses on the motivation, and the conclusions are controversial. Therefore, the reasons for the dispute need to be further analyzed. Moreover, research has mainly focused on the green investment of enterprises, lacking any analysis and evaluation on green investment efficiency.

### Influence of environmental regulation on green investments

As an important means to coordinate social development and environmental protection, environmental regulation has played an important role in environmental governance in recent years by effectively guiding the environmental behavior of enterprises and individuals. Studies mainly measure environmental regulation by the cost of pollution control, capital expenditure on pollution reduction^18^, pollutant discharge^19^, environmental tax burden^6,20,21^, and so on. Based on the differences in the above measurement standards, the impact of environmental regulations on green investment can be divided into three perspectives.

Traditional economists have argued that environmental regulations inhibit firms' green investments. They believe that environmental regulation inhibits firms' green investment. This group of scholars argues that environmental regulation can inhibit green investment by imposing unnecessary costs on firms and having a crowding-out effect on investment in innovation, which reduces the productivity of firms^22–24^. However, some scholars, led by Porter, have challenged that view. Porter believes that environmental regulation can bring “innovation compensation effect”, which is conducive to the realization of the enterprise's environmental performance and economic performance of the joint improvement^25^. Therefore, this group of economists point out that environmental regulation will stimulate enterprises to break the inherent business model and product structure, and put pressure on enterprises to consider environmental issues, so as to realize energy saving and emission reduction^26,27^. In addition, a small number of scholars, based on the “factor endowment hypothesis”, believes in a non-linear relationship between environmental regulation and corporate green investment.

No matter using regression analysis or the SBM-DDF model, studies have shown an inverted U-type non-linear relationship between environmental regulation and corporate green investment^3,28^. In the early stage of strengthening environmental regulations, enterprises increase green investment and change production methods due to legal requirements. However, with increasing environmental regulation, there will be an inflection point in the factor endowment advantage, when the costs of green investment outweigh the benefits of complying with environmental regulations. This is also contrary to the Poter hypothesis, where high pollution control costs lead to "crowding out" of R&D investment^29^, and firms prefer to accept penalties for non-compliance rather than make more green investments.

Most of the literature acknowledges the significant impact of environmental regulation on enterprises’ green investment, but due to differences in indicators and methods adopted by each research institute, the conclusions are inconsistent. A higher level of environmental tax burden closely relates to strict environmental regulations^30^. As an economic means, an environmental tax burden can regulate the environmental behavior of thermal power enterprises by directly affecting their production and operation. Therefore, this study uses environmental tax burden to measure environmental regulation and studies its impact on the green investment efficiency of China’s thermal power enterprises. Doing so provides a theoretical basis for the government to formulate relevant policies for the sustainable development of thermal power enterprises.

### Green investment efficiency

As green investment efficiency has gradually become a crucial factor affecting the sustainable development of energy enterprises, empirical research based on DEA theory has also been widely used by scholars. Some scholars have analyzed the relationship between enterprise green investment and performance by the DEA method and panel vector autoregression method (VAR). Findings show that enterprises’ green investment inhibits productivity improvement^31^. From the perspective of environmental regulation, some scholars have introduced the learning curve theory into the traditional DEA model to describe the dynamic changes of power enterprises under different policy scenarios. The results show an interactive relationship between environmental regulation, environmental protection investment, and the sustainable development of power enterprises^32^. Environmental regulation motivates enterprises to pay attention to environmental performance, thus improving the efficiency of their green investment^33^.

Some scholars hold a different view, with some quantifying the green investment efficiency of heavy polluting enterprises through the SBM-DEA model. The results show an inverted U-shape non-linear relationship between environmental regulation and green investment efficiency of Chinese polluting enterprises^34^. Some scholars have also utilized the Tobit model and found a double effect of environmental regulation on the green efficiency of thermal power enterprises. It is specifically manifested as a U-shape non-linear relationship of first inhibition and then promotion^35^. Therefore, local governments must pay attention to the differentiation of environmental regulations in order to encourage energy enterprises to improve green investment efficiency.

From the perspective of digital empowerment, some scholars have used the SBM-DEA method to conduct quantitative analysis of the green investment efficiency of heavy polluting enterprises. Research has found that digital empowerment promotes green efficiency through increased analyst attention and greater R&D investment^36^.

## Summary

In short, the green investment efficiency evaluation method based on DEA has wide application prospects in the current global low-carbon environmental protection era. These theories also provide a theoretical basis for government departments to guide the environmental management of energy enterprises.

Although there is a growing body of literature on the relationship between environmental regulation, green investment efficiency, and firm performance, there are still some limitations in this research field. In fact, most studies admit under strict environmental regulations that green investment of enterprises positively impacts their performance. However, the existing studies on the relationship between green investment and environmental regulation are relatively broad, mostly focusing on city clusters or all enterprises, and rarely focusing on a particular industry, especially the thermal power industry. As a result, the conclusions and policy recommendations are not fully applicable to all industries, and it is difficult for the government to improve environmental policies accordingly. In addition, the existing literature on green investment at the enterprise level is mainly limited to econometric methods, and there is less literature on the use of DEA models to study the efficiency of green investment in energy enterprises.

## Research method

Based on the fact that the evaluation performance of the DDF non-ray distance function is better and provides more accurate estimation results, this study amends the traditional DDF model, combines the dynamic DEA model of Tone and Tsutsui^37^, and considers the exogenous problem, so as to solve the deficiencies of one-, two-, and three-stage Dynamic DDF under an exogenous DEA model. The formula runs as follows.

Assume that a decision-making unit (${DMU}_{j},j=1,\dots ,J$) has $t (t=1,\dots ,T)$ time periods. Within each time period there are three stages: Stage 1, Stage 2, and Stage 3.

In the first stage, there are D inputs ${x}_{ij}^{t}\left(i=1,\dots ,m\right)$ that produce P intermediate products ${z}_{Pj}^{t}\left(p=1,\dots ,P\right)$ and Q desired outputs ${o}_{qj}^{t}\left(q=1,\dots ,Q\right)$.

The second stage uses P intermediate products ${z}_{Pj}^{t}\left(p=1,\dots ,P\right)$ and F inputs ${a}_{fj}^{t}\left(f=1,\dots ,F\right)$ to create R desired outputs ${y}_{rj}^{t}\left(r=1,\dots ,R\right)$ and S intermediate products ${u}_{sj}^{t}\left(s=1,\dots ,S\right)$.

The third stage uses S intermediate products ${u}_{sj}^{t}\left(s=1,\dots ,S\right)$ and G inputs ${f}_{gj}^{t}\left(g=1,\dots ,G\right)$ to create L desired outputs ${n}_{lj}^{t}\left(l=1,\dots ,L\right)$.

Stage 1 (operation stage) inputs are number of employees and thermal power installed capacity. Output is operating revenue. Stage 1 links to Stage 2 via R&D expenses.

Stage 2 (green investment stage) inputs are green investment, proportion of installed renewable energy capacity, and social donation. Output is the ESG index. Stage 2 links to stage 3 via media attention.

Stage 3 (market performance stage) input is operating costs. Outputs are enterprise market value and market share. Exogenous variable $={A}_{vj}\left(v=1\dots V\right)$ is environmental regulation, and carry-over ${= c}_{hj}^{t}\left(h=1,\dots ,H\right)$ is fixed assets.

Here, $j$ represents the number of each DMU, i.e., the 30 thermal power enterprises in this paper, $t$ represents the stage, and $i,p,q,f,r,s,g,v,h,l$ represent the order of each variable. For example, ${x}_{ij}^{t}$ stands for the *i*’th input of enterprise $j$ in stage $t$.

### Objective function

If there is an n dimension $DMU$ set denoted as *j*, where ${DMU}_{o}$ represents the $DMU$ under evaluation and ${DMU}_{o}\in j,$ then the mathematical model is formulated as follows.

Formula (1) calculates the efficiency of ${DMU}_{o}$. Of these, formula (1) is primarily referenced to Chiu et al^38^.

1 $$ \max \left( {GFE} \right) = \mathop \sum \limits_{t = 1}^{T} \gamma_{t} \left( {w_{1}^{t} \theta_{1}^{t} + w_{2}^{t} \theta_{2}^{t} + w_{3}^{t} \theta_{3}^{t} } \right) $$

*S.T.*

**Table:** 

Stage 1 (Operation stage)	Stage 2 (Green investment stage)	Stage 3 (Market performance stage)
$\mathop \sum \limits_{j}^{n} \lambda_{j}^{t} X_{ij}^{t} \le x_{io}^{t} - \theta_{1o}^{t} q_{io}^{t} \forall i, \forall t$ $\mathop \sum \limits_{j}^{n} \lambda_{j}^{t} z_{Pj}^{t} \le z_{Po}^{t} - \theta_{1o}^{t} q_{Po}^{t} \forall d,\forall t$ $\mathop \sum \limits_{j}^{n} \lambda_{j}^{t} o_{qj}^{t} \ge o_{qo}^{t} - \theta_{1o}^{t} q_{qo}^{t} \forall k, \forall t$	$\mathop \sum \limits_{j}^{n} \mu_{j}^{t} Z_{pj}^{t} \le z_{po}^{t} - \theta_{2o}^{t} q_{po}^{t} \forall d, \forall t$ $\mathop \sum \limits_{j}^{n} \mu_{j}^{t} y_{ri}^{t} \ge y_{ro}^{t} - \theta_{2o}^{t} q_{ro}^{t} \forall s, \forall t$ $\mathop \sum \limits_{j}^{n} \mu_{j}^{t} u_{sj}^{t} \le u_{so}^{t} - \theta_{2o}^{t} q_{so}^{t} \forall g, \forall t$	$\mathop \sum \limits_{j}^{n} \rho_{j}^{t} u_{sj}^{t} \le u_{so}^{t} - \theta_{3o}^{t} q_{so}^{t} \forall e, \forall t$
		$$\mathop \sum \limits_{j}^{n} \rho_{j}^{t} f_{gj}^{t} \le f_{go}^{t} - \theta_{3o}^{t} q_{go}^{t} \forall b, \forall t$$
		$$\mathop \sum \limits_{j}^{n} \rho_{j}^{t} n_{lj}^{t} \le n_{lo}^{t} - \theta_{3o}^{t} q_{lo}^{t} \forall l, \forall t$$
		$$\mathop \sum \limits_{j}^{n} \mu_{j}^{t} u_{sj}^{t} \le u_{so}^{t} - \theta_{2o}^{t} q_{so}^{t} \forall e, \forall t $$

If there is an n dimension $DMU$ set denoted as $j$, where ${DMU}_{o}$ represents the $DMU$ under evaluation and ${DMU}_{o}\in j.$

Here, GFE represents Global-Factor Efficiency. ${\gamma }_{t}$ is the weight assigned to period t, and ${w}_{1}^{t}$, ${w}_{2}^{t}$, and ${w}_{3}^{t}$ are the weights assigned to Stage 1 efficiency, Stage 2 efficiency, and Stage 3 efficiency, respectively. Therefore, ${w}_{1}^{t}$, ${w}_{2}^{t}$, and ${w}_{3}^{t}$ and $\sum_{t=1}^{T}{\gamma }_{t}=1$.

### Exogenous variable and links of stages

The exogenous variable is formula (2). Of these, formula (2) is primarily referenced to Li et al^39^.

$$ \mathop \sum \limits_{j = 1}^{n} \lambda_{j}^{t} A_{vj}^{t} = A_{vo}^{t} \;\;\forall v\;{\text{ t}} = 1,2 \ldots {\text{T}} $$

$$ \mathop \sum \limits_{j = 1}^{n} \lambda_{j}^{t} c_{hj}^{t} \le c_{ho}^{t} - \theta_{1o}^{t} q_{ho}^{t} \;\;\forall h,\; \;\forall t $$

2 $$ \mathop \sum \limits_{j}^{n} \mu_{j}^{t} = 1,\; \mathop \sum \limits_{j}^{n} \rho_{j}^{t} = 1, \;\;\lambda_{j}^{t} \ge 0 \forall j, \;\;\forall t,\;\; \mu_{j}^{t} \ge 0 \forall j,\;\;\forall t,\;\; \rho_{j}^{t} \ge 0 \forall j,\;\;\forall t $$

Here, $\lambda_{j}^{t} ,\;\mu_{j}^{t} ,\;\rho_{j}^{t}$ denote the weights of the benchmarking for ${DMU}_{o}$ in the first, second and three stages, respectively.

Stage 1 and Stage 2 links are formula (3). Stage 2 and Stage 3 links are formula (4). The two periods of links are formula (5). Of these, formula (3), (4), (5) is primarily referenced to Lu et al^40^.

3 $$ \mathop \sum \limits_{j = 1}^{n} \lambda_{j}^{t} Z_{dj}^{t} = \mathop \sum \limits_{j = 1}^{n} \mu_{j}^{t} Z_{dj}^{t} \;\;\forall d,\;\forall t $$

4 $$ \mathop \sum \limits_{j = 1}^{n} \mu_{j}^{t} u_{ej}^{t} = \mathop \sum \limits_{j = 1}^{n} \rho_{j}^{t} u_{ej}^{t} \;\;\forall e,\;\;\forall t $$

5 $$ \mathop \sum \limits_{j = 1}^{n} \lambda_{j}^{t - 1} c_{hj}^{t} = \mathop \sum \limits_{j = 1}^{n} \lambda_{j}^{t} c_{hj}^{t} \;\;\forall h,\;\;\forall t $$

Overall efficiency, period efficiency, stage efficiency, and period stage efficiency can be obtained from the above results.

### Sub-efficiency values

The sub-efficiency values of the variables in this study are calculated in accordance with the Total-Factor Efficiency (TFE) indicator published by Hu and Wang^41^ via the following equation.

Input variables and good output variables are formulae (6) and (7), respectively.

6 $$ {\text{TFE}} = \frac{{{\text{Target}}\;{\text{input}}}}{{{\text{Actual }}\;{\text{input}}}} $$

7 $$ {\text{TFE}} = \frac{{{\text{Actual}}\;{\text{output}}}}{{{\text{Target}}\;{\text{output}}}} $$

If the value of total factor efficiency is 1, then the efficiency target has been achieved; conversely, it means that there is an excess of inputs or a shortage of outputs, indicating that there is room for improvement.

## Empirical analysis

### Data description

This paper selects data from 30 listed thermal power companies in China from 2018 to 2022. The selection is based on thermal power listed companies that are ranked among the top 30 in market capitalization on the Flush Financial Data Platform as of the end of 2022 and have been listed for more than five years. The abbreviations of the sample companies are in “Appendix A”. Since the green investment path of listed thermal power companies is not unique, different companies illustrate differentiated transformation by combining their own advantages.

Studies vary in their division of green investments. According to the green financial products invested, they can be divided into green credit, green securities, and green insurance^42^. According to the use of green investment project funds, they can be divided into expenditures for environmental pollution control and expenditures for environmental infrastructure construction^43^. As the research object of this paper is thermal power listed enterprises, power generation projects are the key aspects of their main business and green investments. Therefore, this paper classifies the green investments of enterprises into three types according to the energy type of the project by analyzing the important ongoing projects of thermal power enterprises: the transformation of thermal power units, photovoltaic projects and wind power projects (Table 1).

**Table 1:** Green investment types of typical thermal power enterprises.

No	DMU	Classification of green investments		
		Retrofit of thermal power units	Photovoltaic projects	Winder power projects
1	GDGI	√	√	√
2	BNEC	√		
3	SNPC	√		
4	JXGN	√	√	
5	DLPC	√		
6	AHWC	√		
7	HEHL	√	√	
8	YNHC		√	√
9	SJEC			√
10	NMHD			√
11	HNPC		√	√

A new energy project is a national key support project that can effectively reduce the carbon emissions of enterprises. From the viewpoint of power generation cost, the cost of photovoltaic power generation has been reduced to parity and even lower than the cost of thermal power. From the viewpoint of geographical adaptability, wind power projects can be built in cities, suburbs, villages, and coastal areas with strong geographical adaptability. However, the instability of a wind project determines that thermal power units need to be used as a peaking power source to ensure stable operation of the grid. A new energy project construction cycle is longer, and it is difficult to achieve results in the short term. Therefore, thermal power companies still carry out thermal power unit renovation to maintain their stable operation.

Among the enterprises in the sample of this study, there are 25, 18, and 11 carrying out thermal power unit renovation, Photovoltaic projects, and wind power projects, respectively. There are 5 enterprises carrying out all three types of projects at the same time. Finally, 13 enterprises carry out both types of projects at the same time.

**Figure 2:**
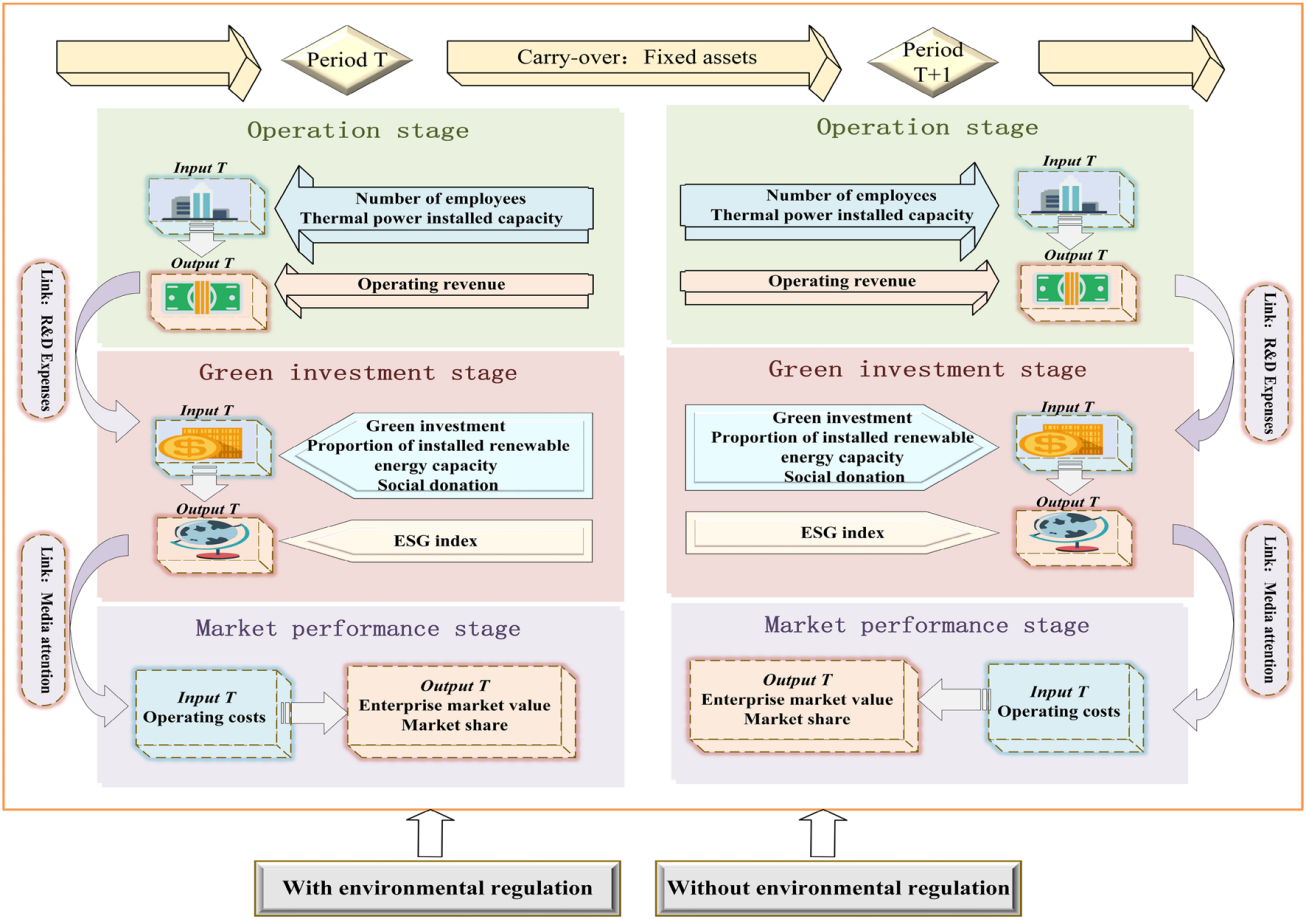
Research framework based on three-stage parallel DEA modeling.

This study evaluates the number of employees, installed thermal power capacity, operating revenue, R&D expenses, green investment, proportion of installed renewable energy capacity, social donations, ESG index, media attention, operating costs, enterprise market value, market share, fixed assets, and environmental regulation of the sample companies from 2018 to 2022. Among them, Min–Max normalization is performed on the raw data of the proportion of installed renewable energy capacity and market share. The research framework based on the three-stage parallel DEA model with relevant indicators is given in Fig. 2.

The main design ideas of the three-stage DEA model in this paper are as follows. First, the process of assessing the green investment efficiency of thermal power enterprises is divided into operation stage (Stage 1), green investment stage (Stage 2), and market performance stage (Stage 3). The first stage is operation. Thermal power enterprises through the normal operation and profitability in this stage lay a good foundation for subsequent green investment. Thermal power installed capacity and the number of employees is mainly selected as inputs, and operating revenue and R&D expenses are outputs. The R&D expenses are continuously invested into the second stage as supportive funds for green investment.

Second, the second stage is green investment. Green investment, the proportion of installed renewable energy capacity, and social donations are taken as inputs. The ESG index and media attention are taken as outputs.

Finally, the third stage is market performance. Media attention is used as an input in the third stage to characterize its important role in a firm’s market performance. Operating costs are an input. Market value and market share are outputs. The T and T + 1 stages are connected through the carry-over variable of fixed assets. Table 2 below specifically explains each indicator.

**Table 2:** Input and output variables meaning.

Stage	Attribute	Variable	Unit	Meaning
Operation stage (Stage1)	Input	Number of employees	Person	Number of employees in the enterprise
		Thermal power installed capacity	10MW	Sum of the rated effective power of the thermal power generator set actually installed in the system
	Output	Operating revenue	10^8^ yuan	Total amount of revenue a business receives in a year from activities
	Link	R&D expenses	10^4^ yuan	Expenses incurred during the research and development of products, technologies, materials, and processes
Carry-over		Fixed assets	10^8^ yuan	Existing value of non-monetary assets held by enterprises for production and operation
Green investment stage (Stage2)	Input	Green investment	10^4^ yuan	Aggregate annual occurrences related to environmental governance, green production, etc. in the schedule of construction in progress
		Proportion of installed renewable energy capacity	%	Ratio of installed renewable energy capacity to total installed capacity
		Social donation	10^4^ yuan	Amount of donations reported in the company’s annual report as non-operating expenses
	Output	ESG index		ESG index from China Securities
	Link	Media attention	Times	Number of web media content occurrences taken from the CNRDS database
Market performance stage (Stage3)	Input	Operating costs	10^8^ yuan	Cost of goods sold or services provided by a business
	Output	Enterprise market value	10^8^ yuan	Product of total share capital and stock price
		Market share	%	Ratio of power generation of enterprises to power generation of the whole industry
Exogenous variable		Environmental regulation	10^4^ yuan	Environmental protection tax paid by enterprises

Data are from annual reports, ESG index, and CNRDS database.

### Descriptive statistics

**Figure 3:**
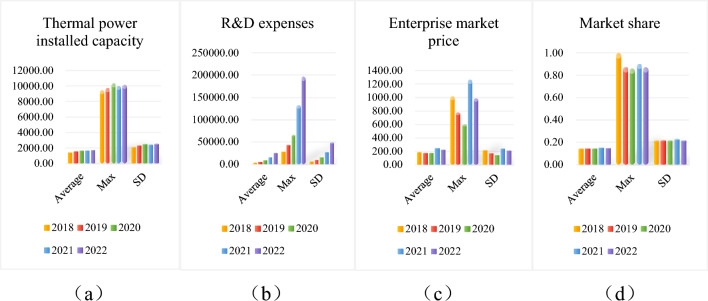
(**a**–**d**) Descriptive statistics of operating and marketability variables.

**Figure 4:**
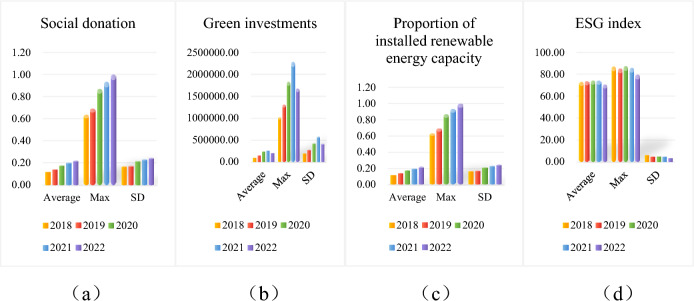
(**a**–**d**) Descriptive statistics of sustainability variables.

Due to space limitation, only major variables are selected for statistical description in this paper. Green investment stage is a key turning point for thermal power enterprises to achieve sustainable development and plays an important role in the steady operation of enterprises. Therefore, the selected indicators are categorized into two types for statistical description herein: operation and market indicators and sustainability indicators. Their mean, maximum, and standard deviation are calculated respectively, and the results are rounded to two decimal places. Figures 3 and 4 show the statistical description of the two categories of variables by year.

In terms of the operation and market performance of thermal power enterprises, their installed capacity (Fig. 3a) has been relatively stable in recent years. R&D expenses (Fig. 3b) have increased year by year, and the growth rate is also increasing year by year. By raising R&D expenses, thermal power enterprises can carry out technological innovation to develop new products or new energy technologies, thus improving their production and operational efficiency. In addition, although the market value of enterprises (Fig. 3c) has obvious fluctuations, the market share (Fig. 3d) is generally stable and does not show large changes.

Looking at the sustainability of thermal power firms, first, social donation (Fig. 4a) is far more volatile than the other variables, with 2019 leading the five-year period in terms of this factor. Second, green investment (Fig. 4b), which had been trending upward in the previous four years, suddenly declined in 2022, possibly due to increased environmental regulations. Finally, the steady growth trend in the proportion of installed renewable energy capacity (Fig. 4c) and the high level of the ESG index (Fig. 4d) reflect that companies are actively pursuing a green and low-carbon transition.

### Empirical result analysis

#### Total efficiency analysis

This study considers the inclusion of exogenous variables and the absence of exogenous variables separately when assessing each DMU. According to the empirical results of this paper, after adding exogenous variables, the green investment efficiencies of 24 out of 30 firms significantly improve. Without considering exogenous factors, the average value of the overall efficiency of enterprises is 0.553, and 10 enterprises have total efficiency greater than 0.6. After considering exogenous factors, the mean value of overall efficiency is 0.591, and 13 thermal power enterprises have total efficiency greater than 0.6. Obviously, environmental regulation as an exogenous variable in the model significantly improves the underestimation of enterprises’ green investment efficiency.

**Figure 5:**
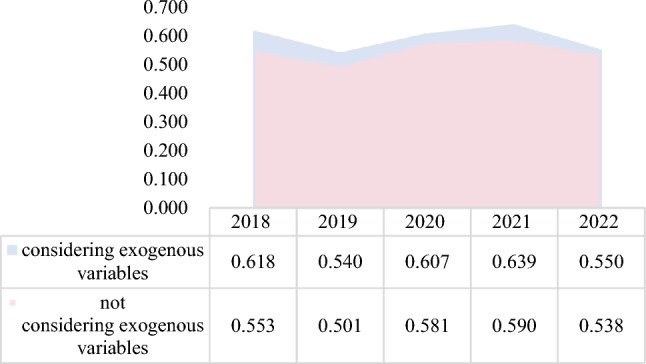
Average green investment efficiency (2018–2022).

As can be seen from Fig. 5, the green investment efficiency of thermal power enterprises was at its lowest in 2019 at 0.501 and hit its peak in 2021 at 0.590. The overall trend is that it first declines, then improves, and finally declines again. In 2018–2019, at the beginning of the strengthening of environmental regulations, the increased cost of thermal power enterprises for pollution control had a crowding out effect on innovation investment. This also led to a decline in the green investment efficiency of thermal power enterprises, from 0.553 to 0.501. With the implementation of environmental policies, enterprises are forced to develop cleaner and more environmentally friendly production methods due to pressure from the environmental tax burden. For example, the renovation of thermal power units and the development of new energy projects spurred the green investment efficiency of enterprises to increase by 8.9% in two years. However, as the proportion of new energy connected to the grid increases, the power system is not flexible enough, and the problem of consumption gradually emerges. The green investment efficiency of enterprises thus exhibits a significant decrease.

Of the 24 thermal power companies that have improved after adhering to the environmental regulation, 18 companies are carrying out retrofitting of thermal power units and 15 are carrying out photovoltaic projects. Relatively fewer firms, less than half, are targeting the construction of wind power projects. Among the top 10 enterprises with the strongest improvement effect, 80% of them have carried out thermal power unit renovation. Obviously, enterprises prefer to realize the improvement of their green investment efficiency by retrofitting thermal power units. On the one hand, retrofitting thermal power units is flexible and has a low cost compared to new energy construction. Thermal power units can operate stably for a long period of time and adjust their output according to power demand. On the other hand, photovoltaic projects and wind power projects have strong volatility, homogeneity and require flexible resource packages to solve the consumption problem. In the case of a significant increase in electricity load, wind and solar clean resources are difficult to provide enough controllable power.

In China there are still provinces with a significant problem of abandoned wind or abandoned light. They include Inner Mongolia, Qinghai, Gansu, and other wind power provinces. Some areas of wind power or photovoltaic power only have a utilization rate of 90%. Therefore, compared with Photovoltaic projects and wind power projects, companies can more feasibly improve their total efficiency through the renovation of thermal power units.

**Figure 6:**
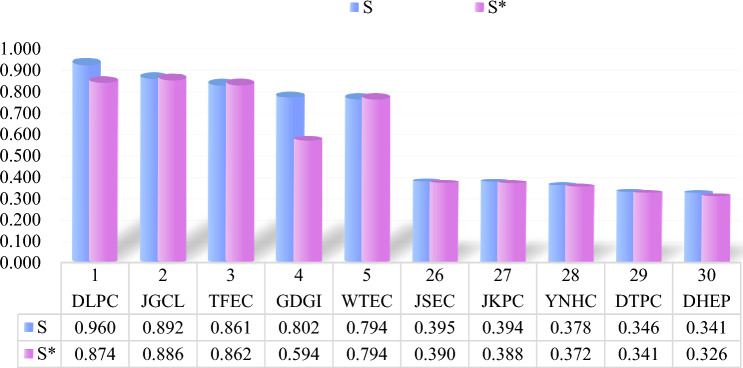
Total efficiency values of thermal power enterprises (top and bottom 5). *Notes*: The ranking is based on the efficiency value after adding exogenous variables. S means considering exogenous variables. S* means not considering exogenous variables.

By comparing the green investment efficiency of the top 5 and bottom 5 thermal power enterprises, this paper finds that efficiency significantly improves after considering exogenous variables. As seen from Fig. 6, the green investment efficiency value of each thermal power enterprise varies greatly. Among them, DHEP has the lowest green investment efficiency value of 0.341, which is only 1% higher than the case without considering environmental regulation. DLPC has the highest efficiency value of 0.960, which is an increase of nearly 10% over the case without taking into account exogenous factors. The improvement is significant. Therefore, the impact of environmental regulations on DLPC is much greater than that on DHEP. This may be due to the fact that DHEP is higher than DLPC in terms of enterprise size, capital cost, and technical feasibility. Its investment in green innovation has already reached a high level, and so the improvement rate is not as high as DLPC. In addition, DHEP’s fuel costs increased by U$$2.953 billion year-over-year in 2021 due to the increase in the unit price of standard coal used for power generation. The significant increase in operating costs leads to a company’s lower gross margin and continued losses. In turn, the focus on green investments continues to diminish, and the efficiency of green investments is bound to decrease.

In terms of the magnitude of improvement, the impact of environmental regulation on GDGI ranks first among the sample firms, with a 20.8% improvement in green investment efficiency. On the one hand, GDGI's earnings continue to be high, and there is enough capital to build on green investments. On the other hand, environmental regulations have been strengthened, and significant results have been achieved in green investments. Therefore, a company can decide to continue to invest in green investments in the future and continue to invest in research and development. From thermal power generation to multiple energy sources, GDGI has always adhered to the direction of clean energy development. The improvement of its green investment efficiency is also an inevitable trend.

In conclusion, environmental regulations have a more significant impact on the green investment efficiency of Chinese thermal power companies. During the period from 2018 to 2022, the average value of the environmental tax burden paid by enterprises is generally increasing. Environmental regulations force thermal power enterprises to improve their production and operation methods by increasing their environmental management costs. Moreover, thermal power enterprises combine their own advantages to carry out green transformation of industrial structure. While realizing green and high-quality development, they also promote the improvement of green investment efficiency.

#### Stage efficiency analysis

##### Efficiency analysis of operation stage (Stage 1)

In the operation stage, this study includes the number of employees, installed thermal power capacity, and operating revenue in the input–output index system of this stage. According to the empirical results of this paper, during the period from 2018 to 2022 the efficiency of thermal power enterprises in the operation stage performs well with an overall mean value of 0.613. However, there are still some enterprises with low efficiency in this stage, such as DTPC, whose efficiency value fluctuates around 0.2 or less than one-third of the average value.

**Figure 7:**
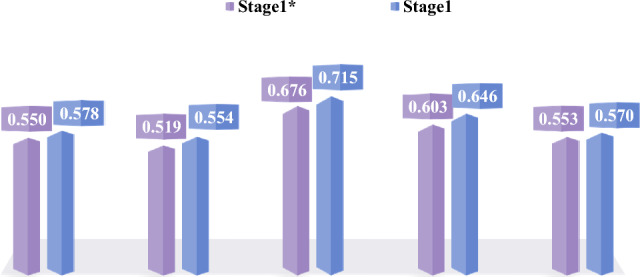
Average value of efficiency in operation stage (2018–2022). *Notes*: Stage1 means considering exogenous variables. Stage1* means not considering exogenous variables.

As seen from Fig. 7, the inclusion of environmental regulation can significantly increase the efficiency of thermal power enterprises in the operation stage. The collection of environmental taxes raises the production costs of highly polluting enterprises, thus promoting the optimal allocation of resources and industrial restructuring. For thermal power enterprises, the collection of environmental taxes pushes them to pay more attention to the development of clean energy and to increase investment in renewable energy. By optimizing the industrial structure, the energy efficiency of thermal power enterprises will improve. In response to the role of exogenous variables in this stage, BNEC is most strongly affected, mainly because BNEC is driven by environmental regulations and focuses on the level of cost optimization and energy savings. It is a commitment to operational efficiency improvement whose improvement effect is as high as 0.462 in 2019.

After 2020 in the late period of strengthening environmental regulations, the operational efficiency of thermal power enterprises has significantly weakened. The average efficiency fell by 14.5% in two years, perhaps due to the need for thermal power companies to invest more resources and funds in technology upgrades in order to meet more stringent environmental requirements. Therefore, for improving energy efficiency and reducing pollutant emissions, there will be some technical or economic restrictions that result in the decline of efficiency at this stage. In summary, environmental regulations can, to some extent, lead to higher efficiency values by inducing improvements in energy use efficiency and reductions in pollutant emissions during the operational stage of an enterprise. However, with increased pressure on profitability, thermal power companies may encounter some constraints in this regard, leading to a slowdown or decline in the rate of improvement of efficiency values.

##### Efficiency analysis of green investment stage (Stage 2)

The green investment stage is an indispensable part of thermal power enterprises to realize sustainable development. This study includes green investment, the proportion of installed renewable energy capacity, social donations, and the ESG index into the input–output index system of this stage. Table 3 shows the efficiency value of green investment stage of thermal power enterprises selected by this paper with more typical performance in this stage.

**Table 3:** Efficiency value of green investment stage of typical thermal power enterprises.

No	DMU	Stage2* 2018	Stage2 2018	Stage2* 2019	Stage2 2019	Stage2* 2020	Stage2 2020	Stage 2* 2021	Stage 2 2021	Stage 2* 2022	Stage 2 2022
1	BNEC	0.263	1.000	0.205	1.000	0.132	0.264	0.532	0.377	0.039	0.035
2	CEPC	0.055	0.072	0.049	0.087	0.438	0.032	0.056	0.183	0.026	0.033
3	DLPC	1.000	1.000	0.938	1.000	1.000	1.000	1.000	1.000	1.000	1.000
4	DTPC	0.075	0.107	0.020	0.046	0.001	0.007	0.074	0.060	0.022	0.020
5	JXGN	0.242	1.000	0.462	1.000	0.210	0.355	0.033	0.232	0.006	0.020
6	GDGI	0.999	1.000	0.831	1.000	0.944	1.000	0.072	1.000	0.041	0.058
7	HDPC	0.059	0.066	0.014	0.028	0.088	0.080	0.169	0.162	0.052	0.042
8	HDEC	1.000	1.000	1.000	1.000	1.000	1.000	1.000	1.000	1.000	1.000
19	JEIC	1.000	1.000	0.337	0.277	0.003	0.034	1.000	1.000	1.000	1.000
10	JGCL	1.000	1.000	0.456	0.509	1.000	1.000	1.000	1.000	1.000	1.000
11	TFEC	1.000	1.000	1.000	1.000	1.000	1.000	1.000	1.000	0.230	0.089
12	TECL	1.000	1.000	1.000	1.000	0.358	1.000	1.000	1.000	1.000	1.000
13	GEPC	0.045	0.047	0.004	0.020	0.125	0.010	0.072	0.036	0.091	0.112

Stage2 means considering exogenous variables. Stage2* means not considering exogenous variables.

The inclusion of environmental regulations has somewhat increased the efficiency value of the green investment stage of thermal power companies during the period from 2018 to 2022. This may be due to the fact that environmental regulation incentivizes firms to approach the green investment business in a more prudent and proactive manner, which in turn promotes green investment efficiency. However, nearly one-third of the thermal power firms, such as HDEC and TFEC (Table 3), have reached the optimal efficiency value before the addition of the exogenous variables, and thus there is no significant improvement.

**Figure 8:**
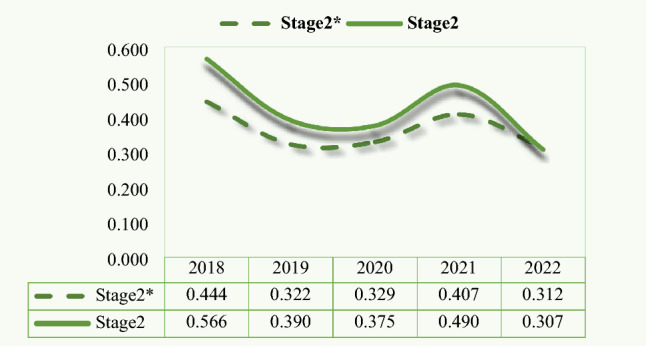
Average efficiency of green investment stage (2018–2022). *Notes*: Stage2 means considering exogenous variables. Stage2* means not considering exogenous variables.

With the increase of environmental regulation pressure, the efficiency value of thermal power companies in the green investment stage shows a large decline. It falls from 0.566 in 2018 to 0.307 in 2022 or a drop of 25.9% (Fig. 8). Even though there is a slight rebound during the period, it again shows a more substantial fall in the following year. This is mainly due to the thermal power enterprises in the process of accelerating the development and utilization of clean energy illustrating that new energy generation will have difficulties in sending out and consuming. The wind turbines and photovoltaic equipment in many areas have been left idle for a long time.

The phenomenon of abandoning light and wind is serious. Moreover, supply chain issues and downward economic pressure due to COVID-19 could have also hindered green investments by thermal power companies between 2021 and 2022. However, some companies have bucked the trend. For example, GEPC’s green investment stage efficiency grew from 0.047 to 0.112, or an improvement of nearly three times.

Compared with the other two stages, the green investment stage has the lowest average efficiency value, between 0.3 and 0.6, and the largest difference in efficiency between enterprises. This is reflected in the serious bifurcation of the efficiency values of the sample enterprises in this stage. The difference between high-efficiency and low-efficiency enterprises is even close to 100%, which is also directly linked to the green investment focus of thermal power companies. Retrofitting thermal power units not only has greater potential and lower investment costs, but also smoothes out the impact of new energy power on the grid.

Thermal power units have a stronger role in improving the efficiency of the green investment stage. For example, JGCL’s green investment projects during this five-year period were dominated by the renovation of thermal power units and supplemented by the construction of new energy projects (Table 3). Thus, JGCL has always maintained a high level of efficiency in the green investment stage. By contrast, HDPC focuses on the construction of new energy projects, such as photovoltaic projects and wind power projects. Therefore, its efficiency in this stage is lower (Table 3).

##### Efficiency analysis of market performance stage (Stage 3)

**Figure 9:**
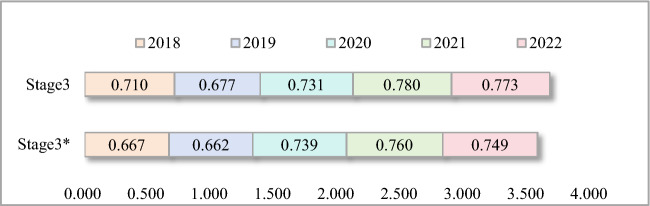
Average efficiency of market performance stage (2018–2022). *Notes*: Stage3 means considering exogenous variables. Stage3* means not considering exogenous variables.

Thermal power firms have the highest efficiency values in the market performance stage compared to the efficiency values in the other two stages. There is also not much difference in the efficiency values among the firms. Except for a few firms such as JNPC, NMHD, and SNPC, most of the other firms have efficiency values fluctuating around 0.7 in this stage. At this stage, the inclusion of environmental regulations had a relatively small effect on improving efficiency, with most firms improving by less than 5%. Overall, the market performance stage efficiency increased year-on-year with the strengthening of environmental regulations and rose much higher than the operational stage (Fig. 9). The unusual performance in 2018 was due to the multiple adjustments in the domestic refined petroleum product market along with the increase in coal prices during the year. They had a dampening effect on the efficiency of most thermal power companies, especially JGCL, in the market performance stage.

The collection of environmental taxes will prompt thermal power enterprises to increase their investment in and development of clean energy. Thus, it will guide enterprises toward restructuring, transformation, and upgrading. For example, GDGI has not only raised its R&D investment year by year, but also continued to increase mergers and acquisitions of high-quality assets and clean energy to optimize its industrial layout.

There are some firms that are not sensitive to environmental regulation. For example, DTPC and HNPC, whose efficiency values in the market performance stage did not improve more significantly from the environmental tax burden (Table 4). The pressure of environmental regulations burden increases the cost of highly polluting and inefficient thermal power firms. For firms adopting clean energy and high efficiency technologies, their costs will be relatively lower. This leads to the exit of inefficient thermal power firms from the market. In turn, high-efficiency firms have the opportunity to expand their market share and increase their efficiency value in the market performance stage.

**Table 4:** Efficiency value of market performance stage of typical thermal power enterprises.

No	DMU	S* 2018	S 2018	S* 2019	S 2019	S* 2020	S 2020	S* 2021	S 2021	S* 2022	S 2022
1	DTPC	0.698	0.698	0.712	0.712	0.785	0.785	0.790	0.790	0.826	0.825
2	GDGI	0.582	0.582	0.581	0.581	0.848	0.770	0.687	0.666	0.662	0.661
3	HNPC	0.582	0.582	0.581	0.581	0.848	0.770	0.687	0.666	0.662	0.661
4	JGCL	1.000	1.000	0.704	0.746	1.000	1.000	1.000	1.000	1.000	1.000
5	JNPC	1.000	1.000	1.000	1.000	1.000	1.000	0.902	0.902	0.925	0.925
6	NMHD	1.000	1.000	1.000	1.000	0.984	0.984	1.000	1.000	1.000	1.000
7	SNPC	0.669	1.000	1.000	1.000	1.000	1.000	1.000	1.000	1.000	1.000

S means considering exogenous variables. S* means not considering exogenous variables.

#### Sub-index efficiency analysis

Due to space limitations, only the main indicators are selected for sub-indicator efficiency analysis in this paper. Table 5 reflects the average values of the efficiency of the main indicators of Chinese thermal power enterprises from 2018 to 2022 with and without considering exogenous variables.

**Table 5:** Average efficiency of key indicators (2018–2022).

	Thermal power installed capacity efficiency		Enterprise market value efficiency		Green investment efficiency		Social donation efficiency		ESG index efficiency	
	S*	S	S*	S	S*	S	S*	S	S*	S
2018	0.685	0.663	0.772	0.931	0.295	0.595	0.372	0.520	0.697	0.907
2019	0.600	0.629	0.838	0.841	0.319	0.398	0.281	0.354	0.751	0.810
2020	0.685	0.730	0.772	0.818	0.295	0.438	0.372	0.352	0.697	0.663
2021	0.636	0.703	0.779	0.826	0.423	0.542	0.384	0.424	0.829	0.732
2022	0.448	0.475	0.743	0.619	0.298	0.274	0.320	0.348	0.714	0.486

S means considering exogenous variables. S* means not considering exogenous variables.

First, the efficiency of installed thermal power capacity directly affects the power generation capacity and capacity level of the enterprise. Hence, it is an important factor for thermal power enterprises to maintain normal business management. After considering the exogenous variable of environmental regulation, the average value of thermal power firms’ installed capacity efficiency increases in most years and peaks at 0.730 in 2020 (Table 5). A few firms are insensitive to the role of environmental regulation, such as JKPC and JNPC. Most companies show a very clustered thermal power installed capacity efficiency of 0.5 or more from 2018 to 2022. For highly polluting and inefficient thermal power units, environmental regulation will increase the operating costs of thermal power enterprises. Therefore, companies will actively adopt energy saving and emission reduction measures and emphasize technological innovation, such as vigorously renovating thermal power units and developing clean energy projects. For example, SNCL has invested in a number of hydrogen energy projects, driven by environmental regulation.

Second, enterprises’ market value efficiency stays at a high level from 2018 to 2021, yet declines substantially in 2022, as in the case of AHWC and GEPC. The inclusion of environmental regulations improves the average market value efficiency of thermal power firms in all years except 2022. In particular, in 2018 the average market value efficiency improves by as much as 15.9%. Environmental regulations can incentivize firms to adopt more environmental protection measures to reduce pollutant emissions, such as ultra-low emissions and desulfurization and denitrification technologies. However, in the late stage of environmental regulation strengthening, the obstruction of new energy construction and excessive cost burden can inhibit the market value efficiency of enterprises. Their average value of efficiency decreases by two-thirds from 0.931 in 2018 to 0.619 in 2022 (Table 5). For example, in 2018 to 2021, DTPC and DLPC have reached the optimal point of their enterprises’ market value indicator efficiency in yearly increments under the pressure of the environmental regulation. In 2022, however, excessive cost burdens led to a significant decline in firm market efficiency, or far less than half of what it was in 2021.

Third, with the strengthening of environmental regulations, the average green investment efficiency value of thermal power enterprises has been declining in fluctuation over the five-year period. The average green investment efficiency value is the highest in 2018 at 0.595, while 2022 has the lowest efficiency at 0.274 or less than half of that in 2018 (Table 5). Green investment efficiency rebounds considerably in 2021, but then deteriorates sharply in the following year, going from 54.2 to 27.4%. SEPC and GDGI have seen a typical plunge in the efficiency of their green investments, with a decline of almost 100%. This is mainly due to the fact that companies pay more attention to compliance in order to avoid being fined or facing other legal risks. This ultimately results in companies not being able to fully utilize the benefits of green investment.

After adding exogenous variables, the average green investment efficiency of the 30 thermal power companies has improved in the previous four years. Especially for BNEC, the addition of environmental regulation directly doubles its efficiency value several times to reach optimal efficiency. During this period, the average social donation efficiency value has fluctuated slightly, but overall, it is still slowly decreasing. The average social donation efficiency value in 2018 ranked first among the five years at 0.520 (Table 5). It is worth noting that both green investment efficiency and social donation efficiency of thermal power enterprises are bifurcated. High-efficiency enterprises, such as DLPC and HDEC, achieve an optimal efficiency of 1 for both green investment efficiency and social donation efficiency. However, more than half of the enterprises have both efficiencies of almost 0. This phenomenon may relate more to the enterprises’ view of social responsibility and their own operation situation.

Finally, the ESG index efficiency of most thermal power companies is decreasing year by year, from 0.907 to 0.486. This is mainly due to the fact that companies will reduce the financial pressure from an environmental tax burden by lowering environmental protection investment, which leads to the inability to fully utilize the benefits of ESG index efficiency. In the first two years, the inclusion of exogenous variables can improve the average ESG index efficiency of thermal power enterprises. However, in the latter three years, environmental regulation shows an inhibitory effect on it, and the inhibitory impact increases year by year. In 2022 the inhibitory effect of environmental regulations on the ESG index efficiency of thermal power companies hit 22.8%. This causes the average ESG index efficiency of firms to fall from 0.714 to 0.486 (Table 5). However, there are also firms that show an abnormal rise. For example, CEPC’s ESG index efficiency has been positively affected by environmental regulation with a small increase in efficiency values.

#### Regression analysis

8 $$ Green\;investment_{it} = \alpha_{0} + \alpha_{1} Environmental\;regulation_{it} + \gamma_{t} + \varepsilon_{it} $$

where *i* denotes the individual company,* t* denotes the year, ${Green investment}_{it}$ denotes the amount of green investment of the thermal power enterprise, ${Environmental\; regulation}_{it}$ denotes the environmental tax burden paid by the thermal power enterprise, ${\gamma }_{t}$ denotes the time fixed effect, and ${\varepsilon }_{it}$ is the random perturbation term.

In order to further verify the impact of environmental regulation on green investment, this paper carries out a benchmark regression for formula (8). The regression results show that environmental regulation is significantly positive at the 1% level, indicating that environmental regulation can promote the green investment of thermal power enterprises. On the one hand, under the pressure of environmental regulation, thermal power enterprises will actively make green investment for tax incentives. On the other hand, environmental regulation will increase the cost pressure on thermal power enterprises, which will encourage them to make green investment to reduce their pollutant emissions. This result further confirms the accuracy of the above DEA model evaluation (Table 6).

**Table 6:** Regression analysis.

	Green investments
Environmental regulation	0.783***
	(7.775)
_cons	4.551***
	(5.700)
N	137
R^2^	0.322
Adj R^2^	0.296

*t* statistics in parentheses, * *p* < 0.1, ** *p* < 0.05, *** *p* < 0.01.

## Conclusions and suggestions

### Discussion

The above analysis shows that environmental regulation improves the green investment efficiency of thermal power enterprises. This is similar to the view of some scholars^26,27,33,44^. Environmental regulation improves the green investment efficiency of enterprises by encouraging them to use low-carbon technologies, which have a positive effect in promoting the sustainable development of enterprises. Some studies have also suggested that the relationship between environmental regulation and green investment is nonlinear^45–47^, mainly because the relationship is affected by industry and regional differences. Most thermal power enterprises are polluting enterprises, which are more sensitive to the role of environmental regulation and also bear a higher degree of pressure to spend on environmental protection compared to their own environmental pollution.

With the continuous strengthening of environmental regulation, the green investment efficiency of thermal power enterprises will gradually decrease. This is slightly different from some of the scholars’ research on heavily polluting enterprises^4,34,48^. It may be due to the fact that our study just selects the relevant data of thermal power enterprises within 5 years. Only some of the old power plants in thermal power enterprises are heavy polluters, and so there is some difference for the future trend of green investment efficiency.

Although the results of this study show a correlation between the green investment focus of thermal power enterprises and their green investment efficiency, there may still be some errors. Future research should explore the specific reasons for the changes in green investment efficiency in depth, taking into account the geographical location of thermal power enterprises and the impact of the policy environment. This will provide a more comprehensive analysis for the literature on green investment efficiency.

### Conclusions

This paper draws the following conclusions by evaluating the operational, green investment and market performance efficiencies of 30 listed thermal power companies from 2018 to 2022, and regressing environmental regulation on green investment.

1. In terms of total efficiency, 80% of the sample firms show a significant improvement in total efficiency with the addition of environmental regulations compared to without exogenous variables. Among them, the improvement of GDGI is the most prominent. The addition of environmental regulations can force high-polluting enterprises to emphasize green innovation through the R&D mechanism, which turn plays a positive role in promoting the total efficiency of thermal power enterprises. However, with the addition of environmental regulations, the total efficiency of thermal power firms exhibits a slight decreasing trend between 2018 and 2022. This is associated with cost pressures on firms and the long-term nature of the reduction in green investments.
2. In terms of stage-specific efficiency, more than 60% of thermal power firms’ green investment stages are less efficient as a result of stronger environmental regulations. Compared with the operation and market performance stages, the green investment stage has the lowest efficiency at an overall average value between 0.3 and 0.6. This suggests that the green investment stage is a more difficult stage in the development process of thermal power enterprises and needs to be emphasized by enterprises.
3. In terms of indicative efficiency, there is a serious bifurcation in the green investment efficiency and social donation efficiency of thermal power enterprises. The difference between high-efficiency enterprises and low-efficiency enterprises even reaches 100%. This is strongly linked to the low-carbon transition strategy and operational financial status among thermal power enterprises.
4. In the benchmark regression analysis, environmental regulation is significantly positive at the 1% level, indicating that environmental regulation promotes green investment in electric utilities. This further confirms the accuracy of the DEA assessment.
5. In terms of the green investment focus of thermal power companies, compared to new energy construction, such as photovoltaic and wind power projects, thermal power unit retrofits are more effective in improving green investments in terms of economics and dexterity. Among the top 10 thermal power companies with the strongest improvement effect, eight of them have undertaken thermal power unit retrofits. Thermal unit retrofits are not only cheaper, but can also promote coal power and renewable energy interconnections. Therefore, their effect on energy utilization efficiency and green investment efficiency is more obvious.

### Suggestions

#### Suggestions for thermal power enterprises

First, thermal power enterprises should pay particular attention to green investment in the process of low-carbon transformation. Enterprises should not only target the expansion and upgrading of thermal power units, but also promote mature energy and low-carbon technologies.

Second, thermal power enterprises should achieve differentiated transformation according to their own situation. Due to wind power, photovoltaic and other new energy generation efficiencies are not high, and in the short term they cannot provide reliable power support. Thus, coal power installed capacity also needs to maintain reasonable growth.

Finally, thermal power companies should strengthen information disclosure. Through open and transparent information, investors can understand the enterprise’s green investment situation and the progress of low-carbon transformation as well as enhance investor confidence. In addition, through information disclosure, thermal power enterprises can also learn from the successful experience and lessons of other enterprises in order to improve their own green investment efficiency.

#### Suggestions for government departments

First, government departments should formulate medium- and long-term plans for the transformation of the thermal power industry as early as possible. The formulation of environmental regulations should be coordinated with market operation mechanisms. They can guide thermal power enterprises to form a market competition pattern for green investment. Governments can also provide policy preferences to enterprises that meet environmental protection standards and formulate and implement further incentive policies.

Second, in view of the phenomenon of increasing wind and solar curtailment in some local areas, government departments should coordinate solutions to the large-scale development and high-level consumption of new energy. When implementing unified dispatch across the entire grid, the State Grid Corporation should break through inter-provincial barriers, accelerate the construction of a new power system, and minimize the start-up period of thermal power as much as possible. At the same time, it can implement mandatory consumption of new energy across the entire grid. When wind and solar energy are curtailed, it can obligatorily reduce the output of matched thermal power and prioritize the transmission of new energy.

### Supplementary Information

Supplementary Information.

Supplementary Information 1.

## Data Availability

All data generated or analyzed during this study are included in this published article and its supplementary information files.
