# Supplementary material for: The impact of environmental regulation on green investment efficiency of thermal power enterprises in China-based on a three-stage exogenous variable model
Source: Sci Rep. 2024 Apr 10;14:8400. doi: 10.1038/s41598-024-58396-x (PMC11006837; doi:10.1038/s41598-024-58396-x)
Supplement: Supplementary file 2 — Supplementary Information 1. [file 41598_2024_58396_MOESM2_ESM.docx]

**Appendix A**

Sample business name abbreviation

| **NO.** | **DMU** | **abbreviation** |
| --- | --- | --- |
| 1 | Jointo Energy Investment Co., Ltd. Hebei. | JEIC |
| 2 | Xinjiang Tianfu Energy Co., Ltd. | TFEC |
| 3 | Huadian Power International Corporation Limited | HDPC |
| 4 | Beijing Jingneng Power Co., Ltd. | JNPC |
| 5 | Wintime Energy Group Co., Ltd. | WTEC |
| 6 | Huadian Energy Company Limited | HDEC |
| 7 | Huaneng Power International, Inc. | HNPC |
| 8 | Inner Mongolia MengDianHuaNeng Thermal Power Corporation Limited | NMHD |
| 9 | Shanghai Electric Group Company Limited | SEPC |
| 10 | Datang International Power Generation Co., Ltd. | DTPC |
| 11 | Shenyang Jinshan Energy Co., Ltd. | SJEC |
| 12 | Jinneng Holding Shanxi Electric Power Co., Ltd. | JKPC |
| 13 | Henan Yuneng Holdings Co., Ltd. | YNHC |
| 14 | Jiangsu Guoxin Corp., Ltd. | JGCL |
| 15 | GD Power Development Co., Ltd. | GPDC |
| 16 | Shenergy Company Limited | SNCL |
| 17 | Shenzhen Energy Group Co., Ltd. | SEGC |
| 18 | Guangdong Electric Power Development Co., Ltd. | GEPC |
| 19 | Datang Huayin Electric Power Co., Ltd. | DHEP |
| 20 | Zhejiang Zheneng Electric Power Co., Ltd. | ZEPC |
| 21 | CHN Energy Changyuan Electric Power Co., Ltd. | CEPC |
| 22 | Guangzhou Hengyun Enterprises Holdings Ltd. | HEHL |
| 23 | AnHui Wenergy Company Limited | AHWC |
| 24 | Top Energy Company Ltd. Shanxi | TECL |
| 25 | Dalian Thermal Power Co., Ltd. | DLPC |
| 26 | Jiangxi Ganneng Co., Ltd. | JXGN |
| 27 | Jilin Electric Power Co., Ltd. | JEPC |
| 28 | Shenzhen Nanshan Power Co., Ltd. | SNPC |
| 29 | Guangdong Baolihua New Energy Stock Co., Ltd. | BNEC |
| 30 | Guangzhou Development Group Incorporated | GDGI |
